# No additional prognostic value for MRE11 in squamous cell carcinomas of the anus treated with chemo-radiotherapy

**DOI:** 10.1038/bjc.2017.188

**Published:** 2017-06-22

**Authors:** Alexandra K Walker, Christiana Kartsonaki, Elena Collantes, Judith Nicholson, Duncan C Gilbert, Anne E Kiltie

**Affiliations:** 1 CRUK/MRC Oxford Institute for Radiation Oncology, University of Oxford, Oxford OX3 7DQ, UK; 2 Nuffield Department of Population Health, University of Oxford, Oxford OX3 7DQ, UK; 3 Medical Research Council Population Health Research Unit (MRC PHRU) at the University of Oxford, Oxford OX3 7DQ, UK; 4 Department of Cellular Pathology, Oxford University Hospitals NHS Foundation Trust, John Radcliffe Hospital, Oxford OX3 7DU, UK; 5 Sussex Cancer Centre, Royal Sussex County Hospital, Eastern Road, Brighton BN2 5BE, UK

**Keywords:** anal cancer, human papillomavirus, immunohistochemistry, MRE11, p16, tumour-infiltrating lymphocytes

## Abstract

**Background:**

The majority of anal cancers (84–95%) are driven by infection with human papillomavirus (HPV). HPV-positive tumours show significantly better responses to chemo-radiotherapy when compared with HPV-negative tumours. HPV infection is linked to alterations in DNA damage response proteins, including MRE11. MRE11 is a potential predictive biomarker for response to radiotherapy in muscle-invasive bladder cancer and may hold predictive power in other cancers.

**Methods:**

Using a previously reported cohort, we evaluated the levels of MRE11 in anal cancer and assessed its predictive value in this disease.

**Results:**

We found no association between the level of MRE11 and relapse-free survival following chemo-radiotherapy.

**Conclusions:**

MRE11 has no predictive value in the analysis of relapse-free survival after chemo-radiotherapy in anal cancer and does not add to the prognostic value of p16 and tumour-infiltrating lymphocyte scores. Further investigation into the role of DNA repair proteins in anal cancer is required.

Although anal cancer remains a relatively rare disease, its incidence is increasing (Shiels *et al*, 2015). Most cases (approximately 85%) are squamous cell carcinoma of the anus and anal canal (SCCA) with the remaining cases attributed to adenocarcinoma (10%) or other types (5%) (Nelson *et al*, 2013). Small (T1) tumours may be treated with surgical excision alone, but for most tumours, the standard of care is combination chemo-radiotherapy (Glynne-Jones *et al*, 2009), which achieves good responses with complete tumour regression in 80–90% of cases (Vinayan and Glynne-Jones, 2016). However, acute toxicities resulting from current treatment regimens are common and patients can suffer long-term effects, including bowel, bladder and sexual dysfunction, and more rarely lower limb venous thrombosis (Ghosn *et al*, 2015).

Infection with high-risk subtypes of human papillomavirus (HPV), predominately HPV16, are strongly associated with the development of anal cancer, with reported estimates of HPV positivity in anal cancer ranging from 84% to 95% (Baricevic *et al*, 2015). HPV status, either measured directly or by overexpression of the surrogate marker p16, has a significant effect on patient outcome. Individuals identified as negative for HPV show significantly worse response to therapy compared with patients positive for HPV infection (Gilbert *et al*, 2013; Doll *et al*, 2014; Koerber *et al*, 2014; Serup-Hansen *et al*, 2014; Mai *et al*, 2015; Meulendijks *et al*, 2015), indicating that the current standard treatment is suboptimal for these patients. Further to this, the presence of tumour-infiltrating lymphocytes (TIL) has also been found to stratify p16-positive SCCA. Patients with high TIL scores show significantly better relapse-free survival compared with patients with low TIL scores (Gilbert *et al*, 2016).

The DNA damage response (DDR) is responsible for sensing, signalling and repair of DNA damage. It comprises a network of cellular pathways leading to the activation of a number of cellular mechanisms, including DNA damage repair, cell cycle checkpoints, apoptosis and chromatin remodelling. MRE11 is involved in the initiation of the DDR and functions within the MRN (MRE11-RAD50-NBS1) protein complex (Ciccia and Elledge, 2010). HPV infection is known to activate the DDR system for amplification of the viral genome and recruits DDR proteins to sites of viral replication (Moody and Laimins, 2009; Sakakibara *et al*, 2011; Reinson *et al*, 2013). Additionally, HPV-positive cells also show increased expression of DDR proteins, including MRE11 and other members of the MRN complex (Johnson *et al*, 2017).

MRE11 has been identified as a potential biomarker for outcome following radiotherapy in muscle-invasive bladder cancer. Patients who exhibited high levels of MRE11 showed improved cancer-specific survival following radiotherapy compared with those with low levels (Choudhury *et al*, 2010; Laurberg *et al*, 2012), but no association was seen following cystectomy. Owing to the aberrant activity of the DDR system caused by HPV infection and the high proportion of HPV-driven anal carcinomas, we wished to test the hypothesis that MRE11 might be a useful additional biomarker in anal cancer.

## Materials and methods

With the appropriate ethical approval (11/LO/1032), clinical details and corresponding tumour blocks were retrieved from patients treated with radical chemo-radiotherapy for non-metastatic SCCA from 2004 to 2009 as previously described (Gilbert *et al*, 2013).

Immunohistochemistry was conducted on four-micron thick tissue sections using a Leica Bond-max autostainer (Leica Microsystems GmbH, Wetzlar, Germany) with a Bond Polymer Refine Detection kit (DS9800: Leica Microsystems Inc., Newcastle, UK) and assay-specific reagents. Epitope retrieval was conducted with low-pH buffer for 20 min. A protein blocking step with 10% BSA for 30 min was applied before incubation with MRE11 primary antibody (ab214, Abcam, Cambridge, UK). Primary antibody was used at a 1 : 6000 dilution with samples incubated for 8 min. Leica polymer and postpolymer were applied for 8 min followed by DAB for 10 min and a haematoxylin counterstain for 1 min.

**Figure 1:**
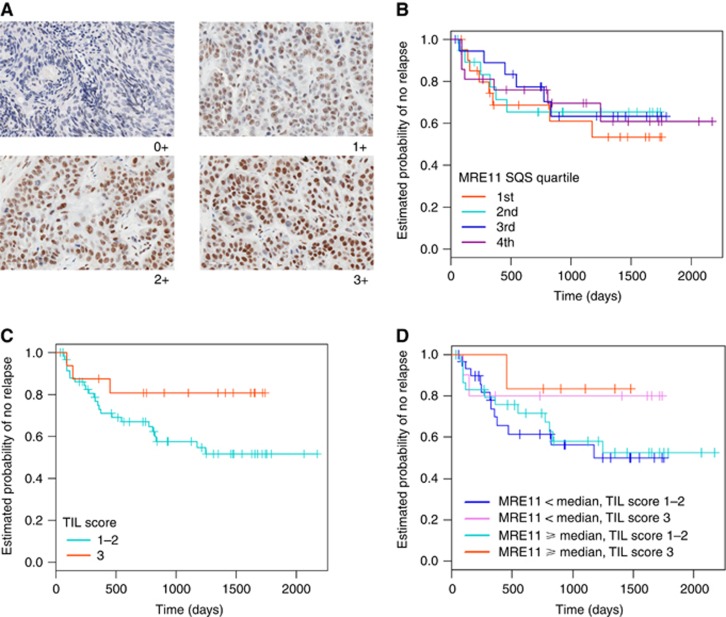
**Results of MRE11 Immunohistochemistry in anal cancer specimens.** (**A**) Representative staining intensities of MRE11 in anal cancer tissue. (**B**) Kaplan–Meier relapse-free survival curves for 82 anal cancer patients stratified by MRE11 semiquantitative score (SQS). MRE11 SQSs are grouped into four quartiles. (**C**) Kaplan–Meier relapse-free survival curves for 78 anal cancer patients stratified by tumour-infiltrating lymphocyte (TIL) score. TIL scores are grouped into low–moderate (1–2) or high (3). (**D**) Kaplan–Meier relapse-free survival curves for 78 anal cancer patients stratified by MRE11 SQS (greater than or equal to and less than the median score) and TIL scores (low–moderate (1–2) or high (3)).

The Aperio ScanScope CS2 digital slide scanner (Leica Microsystems GmbH, Wetzlar, Germany) was used to image the stained sections at × 400 magnification, which were viewed with Aperio Image Scope viewing software (Leica Biosystems Imaging, Vista, CA, USA). Invasive cancerous areas of stained tissue were marked on H&E sections of corresponding tissue by a trained pathologist (EC). Up to 10 high-magnification images of these areas were captured. The intensity and percentage of cancer cells stained for MRE11 were scored in identified invasive cancerous areas by two independent scorers. The intensity score was determined according to a range of 0+ (negative), 1+ (weak), 2+ (moderate) or 3+ (strong staining) (Figure 1A). A consensus score for both intensity and percentage of cancer cells stained in each section was reached and semiquantitative scores (SQSs) were generated (intensity × percentage positive).

Associations between clinicopathological characteristics and relapse-free survival were assessed using multivariable Cox proportional hazards models. Relapse-free survival time was defined as the time from diagnosis of SCCA to the date of diagnosis of confirmed relapse. Individuals were censored at the date of last follow-up. The proportional hazards assumption was checked using scaled Schoenfeld residuals. Statistical analyses were conducted using the statistical software R (R Core Team, 2016).

## Results

Analysis was conducted on a subset of samples from a previously reported SCCA cohort (Gilbert *et al*, 2013). In total, attempts were made to stain tissue from 124 out of 138 individual patients. Loss of patients from the cohort arose due to lack of remaining tissue in block, absence of invasive pathology or presence of cauterisation.

MRE11 immunohistochemistry was then conducted on 124 patients. Forty-two patients were excluded from the analysis due to loss of evaluable tissue. The patient demographic data for the 82 patients’ evaluable samples is shown in Table 1. This patient subset showed the same associations with relapse for p16 and p53 as previously reported (Gilbert *et al*, 2013). In univariate analysis, p16-positive cases showed significantly lower risk of relapse (HR=0.16, (95% CI=0.07–0.40, *P*<0.001)) compared with the p16-negative cases, whereas strong p53 staining was significantly associated with a higher risk of relapse (HR=2.3, (95% CI=1.05–5.22, *P*=0.037)) compared with negative-to-moderate p53 staining.

Univariate analysis of the MRE11 staining, assessed using SQS, showed no association between levels of MRE11 and risk of relapse (HR=0.999, 95% CI=0.994–1.004, *P*=0.61; Figure 1B). In multivariable Cox regression analysis with T stage, N stage, sex, p16 status, p53 status and MRE11 SQS as covariates, only p16 was found to be of prognostic value (HR=0.093, 95% CI=0.024–0.359, *P*<0.001), a result consistent with previously published data on this cohort (Gilbert *et al*, 2013). This result was also seen when multivariate Cox regression analysis was conducted using constituent MRE11 intensity and percentage of positive scores in place of MRE11 SQS (HR for p16: 0.039 (95% CI=0.006–0.230, *P*<0.001), HR per unit increase of MRE11 log(percentage positive): 0.37 (95% CI=0.065–2.080, *P*=0.26), HR for MRE11 intensity 2 *vs* 1: 1.98 (95% CI=0.548–7.175, *P*=0.30) and HR for MRE11 intensity 3 *vs* 1: 0.65 (95% CI=0.128–3.287, *P*=0.60)).

Analysis was attempted on p16-negative cases but the number of p16-negative individuals was too small for reliable statistical analysis. There was no substantial difference in the range of staining observed between p16-positive and p16-negative cases. TIL have recently been reported to add prognostic value over and above p16 immunohistochemistry in SCCA, in part using this cohort of patients (Gilbert *et al*, 2016). We therefore investigated MRE11 alongside TIL scores on this cohort. The addition of MRE11 SQS to TIL score had no significant prognostic value over and above TIL score alone (Figures 1C and D).

**Figure 2:**
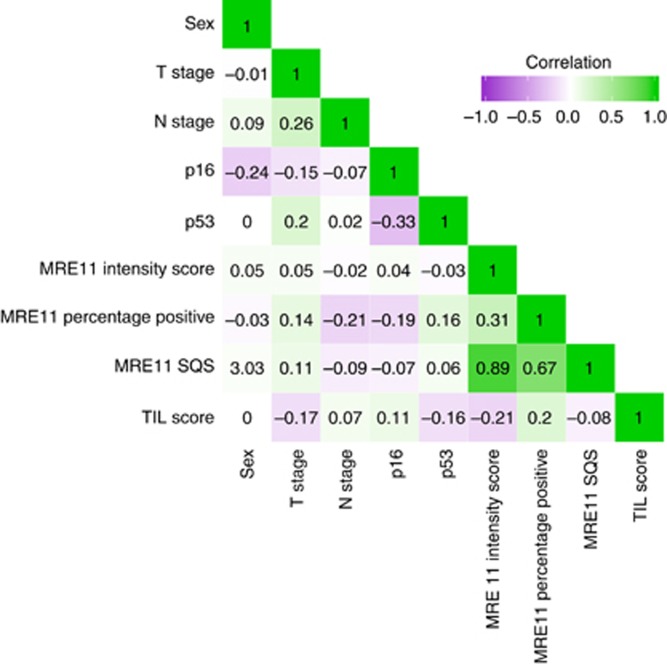
**Correlation matrix for all variables used in the analysis.** Negative values of Pearson’s correlation coefficient, in purple, indicate a negative correlation between variables. Positive values, in green, indicate a positive correlation between variables. The intensity of the purple or green colour indicates the strength of the correlation between the variables.

MRE11 levels, assessed by SQS, percentage positivity or intensity scoring, were not highly correlated with any of the variables used in the analysis (Figure 2).

## Discussion

In contrast to our previous findings in muscle-invasive bladder cancer, we did not identify MRE11 as having predictive value in assessing outcome in SCCA, despite both types of cancer being treated with definitive chemo-radiotherapy. Presumably this represents the heterogeneity that exists between not only different cancers but also different cancer subtypes (Burrell *et al*, 2013). We additionally assessed whether MRE11 had any role to play over and above previously described prognostic markers (p16, p53 and TIL (Jones *et al*, 2017)) but found no evidence of an association with outcome. It is possible that, despite the lack of correlation to outcome in p16/HPV-positive tissue, the assessment of MRE11 levels in p16/HPV-negative tissues might yield positive findings. However, owing to the rarity of the pathology, this would be difficult to investigate.

In SCCA, chemo-radiotherapy achieves a good response rate. However, the toxicities resulting from treatment can be significantly detrimental to patient’s quality of life, and for some patients, particularly those with HPV-negative tumours, outcomes are poor. There is much evidence for alteration in the DDR during HPV transformation (Anacker and Moody, 2017), but this has not yet been fully examined in the context of SCCA and, in particular, its response to therapy. To improve treatment outcomes, further investigation into the molecular diversity of anal cancer is needed.

## Figures and Tables

**Table 1:** Patient demographics for 82 patients whose samples were stained for MRE11

Characteristic	Number of individuals (%)
**Sex**	
Female	43 (52.4)
Male	39 (47.6)
**T stage (*****n*****=68)**	
T1	7 (8.5)
T2	26 (31.7)
T3	22 (26.8)
T4	13 (15.9)
NA	14 (17.1)
**N stage (*****n*****=71)**	
N0	40 (48.8)
N1	9 (11.0)
N2	19 (23.2)
N3	3 (3.6)
NA	11 (13.4)
**p16 (*****n*****=82)**	
p16 positive	72 (87.8)
p16 negative	10 (12.2)
**p53 (*****n*****=82)**	
Strong p53 staining	19 (23.2)
Negative–moderate p53 staining	63 (76.8)
**TIL score (*****n*****=78)**	
TIL 1 (low/absent)	18 (23.1)
TIL 2 (moderate)	44 (56.4)
TIL 3 (high)	16 (20.5)
NA	4 (5.1)
Relapse (*n*=82)	27

Abbreviations: NA=not available; TIL=tumour-infiltrating lymphocyte.
