# No additional prognostic value for MRE11 in squamous cell carcinomas of the anus treated with chemo-radiotherapy

**DOI:** 10.1038/bjc.2017.410

**Published:** 2018-01-11

**Authors:** Alexandra K Walker, Christiana Kartsonaki, Elena Collantes, Judith Nicholson, Duncan C Gilbert, Anne E Kiltie

**Correction to:** *British Journal of Cancer* (2017) **117**, 322–325; doi:10.1038/bjc.2017.188; published online 22 June 2017

This article was published first under a CC-BY-NC-SA licence. The authors have submitted a new licence to make this article accessible under the CC-BY licence. The HTML and online PDF version of this article now carry the updated licence.

